# Effectiveness of Physical Methods in Accelerating Upper Canine Retraction: A Systematic Review and Meta-Analysis of Randomized Controlled Trials

**DOI:** 10.7759/cureus.104768

**Published:** 2026-03-06

**Authors:** Abdulmalek M.H. Almasri, Mohammad Y. Hajeer, Ahmad Othman, Ahmad S. Zakaria, Alaa Oudah Ali Almusawi

**Affiliations:** 1 Department of Orthodontics, Faculty of Dentistry, University of Damascus, Damascus, SYR; 2 Department of Orthodontics, School of Dental Sciences, Universiti Sains Malaysia, Kubang Kerian, MYS; 3 Department of Orthodontics, Faculty of Dentistry, University of Al-Knooz, Basrah, IRQ

**Keywords:** electromagnetic field, low-level laser therapy, orthodontics, tooth movement acceleration, upper canine retraction, vibration

## Abstract

Orthodontic treatments for maxillary canine retraction often extend over long durations, affecting patient comfort and compliance and leading to growing interest in using physical modalities such as low-level laser therapy (LLLT), mechanical vibration, and electromagnetic fields to accelerate tooth movement. This study systematically evaluates and compares the effectiveness of these physical methods in increasing the rate of upper canine retraction in fixed-appliance orthodontic patients.

A comprehensive electronic search of six databases was conducted through July 5, 2025, to identify randomized controlled trials (RCTs) evaluating LLLT, mechanical vibration, or electromagnetic interventions for maxillary canine retraction. Eligible studies compared these modalities with standard orthodontic controls and reported the rate of upper canine retraction (mm/month) as the primary outcome, alongside secondary outcomes including canine angulation, rotation, anchorage loss, and root resorption. Risk of bias was assessed using the Cochrane RoB2 tool, and meta-analyses were performed to calculate mean differences (MDs), p-values, and heterogeneity (I²).

Sixteen RCTs with 322 participants were included, revealing that LLLT significantly increased the retraction rate (MD = 0.43 mm/month, p = 0.007; I² = 97%), which, after sensitivity analysis, became MD = 0.26 mm/month (I² = 48%). Mechanical vibration also significantly increased retraction (MD = 0.36 mm/month, p < 0.00001), with no heterogeneity (I² = 0%). Evidence for electromagnetic fields was limited but still positive. For secondary outcomes, no significant adverse effects were noted for angulation, rotation, anchorage, or root integrity, although one study reported minimal, nonsignificant root resorption.

In conclusion, physical modalities - particularly LLLT and mechanical vibration - can moderately accelerate upper canine retraction without detectable adverse effects. However, due to variability in protocols and limited long-term data, further high-quality RCTs with standardized methodologies are warranted before routine clinical application.

## Introduction and background

Orthodontic treatment is a meticulous and lengthy process, often requiring significant time and patience from patients and practitioners. Long treatment periods can be uncomfortable and inconvenient for patients, often leading to increased physical discomfort and the hassle of frequent dental visits [1]. Additionally, braces and other orthodontic appliances can make it challenging to maintain good oral hygiene, increasing the risk of dental issues such as caries, gingival disease, and white spots on teeth over prolonged periods [2]. Patients who use braces or aligners for a long time may experience a decline in self-esteem. This is yet another reason why shorter treatment times are more useful from a psychological perspective [3]. Shorter treatment durations are beneficial from an economic standpoint as well, as they minimize the total cost of care by reducing the need for multiple visits, adjustments, and corrective procedures [4]. Furthermore, patients - especially younger ones - may find it difficult to adhere to the treatment plan over a long period, so shortening the treatment time can improve compliance and ensure better outcomes. Ultimately, faster treatment may lead to more efficient and effective outcomes, enhancing the overall success rate of orthodontic procedures and significantly improving patient satisfaction and well-being [5,6].

The extraction of the first bicuspids is the most common procedure for camouflaging Class II malocclusion and treating bimaxillary protrusion. This procedure allows for retraction closure in one or two stages. Two-stage retraction, or two-phase retraction, is when the continual retraction of teeth is performed in two discrete steps: the first phase involves the retraction of the canines, and the second stage is the retraction of the incisors [7,8]. An important phase of orthodontic therapy is the retraction of the upper canines, as they affect occlusal and tooth harmony. Given its long duration, there is growing interest in methods designed to accelerate tooth movement during this phase, and shorten the overall therapy period [9,10].

Numerous methods have been developed to facilitate tooth movement through various types of surgeries, both invasive and less invasive, and some even offer less invasive approaches, which remains a controversial topic regarding patients’ acceptance of the methods [11]. Regarding non-surgical approaches, the use of ultrasound to accelerate orthodontic movement has attracted significant attention over the past several years. Some of these include vibration devices [12], low-level laser therapy (LLLT) [13], pulsed electromagnetic fields (PEMFs) [14], and low-intensity electrical currents [15], which have shown promise in preclinical and clinical studies. All of these methods aim to enhance biological activities at the cellular level of bone remodeling and tooth movement, thereby reducing treatment time and improving patient comfort and acceptance.

A review of studies on the physical acceleration of maxillary canine retraction reveals inconsistent findings. In a split-mouth study, Abd-El-Ghafour Omar et al. applied LLLT weekly and biweekly. However, the study found no significant difference in retraction rate between the treated side and the control [16]. In contrast, Mohamed Hasan et al. used a slightly different LLLT protocol and observed a significant increase in tooth movement during the first four months [17]. Testing the AcceleDent Aura device, Abd ElMotaleb et al. reported no difference when it was used for 20 minutes daily [18]. However, Pavlin et al., using the same device, reported a 48% increase in the retraction rate [19]. Alqaisi et al. applied a static magnetic field (SMF) via an auxiliary wire with a magnet, and they recorded significant acceleration compared to the control side, with rates of 23.7% [14]. These studies highlight variability in outcomes, depending on the technique and protocol used.

Different studies yield varied results, underscoring the need for a comprehensive review of the impact of physical methods on the acceleration of upper canine retraction. Many reviews focus on specific techniques, such as LLLT, mechanical vibrations, and electromagnetic fields, but none have examined all of these methods in the context of upper canine retraction. Previous reviews were limited by narrow scope (e.g., focusing on a single modality), small sample sizes, or insufficient emphasis on the canine retraction phase. For instance, Ge et al. [20] and Bakdach and Hadad [21] analyzed LLLT without comparison to other techniques, while Pascoal et al. [22] investigated vibrations but excluded lasers and electromagnetic fields. A recent review by Alam et al. [23] examined the use of electromagnetic fields to accelerate orthodontic tooth movement; however, it did not specifically target upper canine retraction.

This review addresses the gap left by other studies by systematically analyzing LLLT, mechanical vibrations, and electromagnetic fields, answering a question others have not addressed: how effective are physical methods in accelerating the retraction of upper canines?

## Review

Materials and methods

Scoping Search

Before initiating this systematic review, a comprehensive PubMed search was conducted to confirm the absence of prior systematic reviews on the same topic, and to identify potentially eligible trials. The search results revealed no existing systematic reviews addressing this specific topic and identified seven randomized controlled trials (RCTs) that were potentially eligible for inclusion. This report was prepared following the Preferred Reporting Items for Systematic Reviews and Meta-Analysis (PRISMA) guidelines [24]. The systematic review was also registered in the PROSPERO database under registration number CRD420251073202.

Eligibility Criteria

The eligibility criteria were formulated using the Population-Intervention-Comparator-Outcomes-Study design (PICOS) methodology. The target population included patients aged 12-40 years with malocclusions that indicated the extraction of the upper first premolars, followed by a two-step retraction of the upper anterior teeth. The intervention involved applying one of the physical acceleration methods (LLLT, vibrations, and electromagnetic fields). The comparison group was similar to the intervention group, except that the acceleration intervention was not applied. The outcome of interest was the rate of upper canine orthodontic tooth movement, canine rotation, tipping, root resorption, and anchorage loss. All included studies were RCTs, with either a split-mouth or parallel-group design. The inclusion and exclusion criteria for study selection are elaborated in Table 1.

**Table 1:** Inclusion and exclusion criteria according to the PICOS framework PICOS: population, intervention, comparator, outcomes, and study design; LLLT: low-level laser therapy

PICOS framework	Inclusion criterion	Exclusion criteria
Population	Patients aged 12-40 years; the presence of a malocclusion that indicated the extraction of the upper first premolars, followed by a two-step retraction of the upper anterior teeth.	Patients younger than 12 years or older than 40 years. Cases treated with en masse retraction of upper anterior teeth.
Intervention	The application of one of the physical acceleration methods (LLLT, vibrations, and electromagnetic fields).	The application of other methods of acceleration (i.e., surgical, biomechanical, pharmacological, or biological substances).
Comparator	A similar group without the application of the acceleration intervention.	N/A
Outcomes	The rate of upper canine retraction; the rate or amount of canine rotation, tipping; the magnitude of root resorption; the magnitude of anchorage loss.	Studies that did not evaluate the previous outcomes and focused only on patient-reported outcome measures, quality of life, or harms other than root resorption.
Study design	Randomized controlled trials (RCTs) with any design (split-mouth or parallel-group designs).	Non-randomized controlled clinical trials; Cohort studies, Case-control studies, Cross-sectional studies, Case reports, Case series reports, Editorials, Commentaries, Technical notes.

Search Strategy

The digital literature search was conducted using the following databases: PubMed®, Scopus®, EMBASE®, the Cochrane Central Register of Controlled Trials, Web of Science™, and Google Scholar for all studies published up to July 5, 2025. The keywords used in the electronic search are given in Table 2, whereas the details of the search strategy are presented in Appendix 1.

**Table 2:** Keywords used in the search strategy

Aspect of the search query	Keywords
Orthodontics or type of malocclusion	Orthodontic treatment, orthodontic therapy, orthodontics, tooth movement, orthodontic tooth movement, tooth displacement, class II malocclusion, class II division I, class II division II, bimaxillary protrusion.
Type of orthodontic tooth movement or source of orthodontic force	Canine retraction, canine distalization, two-step retraction, space closure, extraction therapy, close coil spring, and power chain elastic.
Acceleration method	Acceleration, tooth movement acceleration, rapid, velocity, duration, rate, regional accelerated phenomenon (RAP), low-level laser therapy (LLLT), low-intensity laser therapy (LILT), pulsed electromagnetic field (PEMF), static electromagnetic field (SMF), mechanical vibrations, vibrational force, light-emitting diode-mediated photobiomodulation therapy (LPT).

Study Selection and Data Extraction

The two review authors (AMHA and AOO) independently evaluated the articles for eligibility. When a disagreement arose, the third author (MYH) was asked to resolve the matter until an agreement was reached. When questions arose, or further clarification was required, the authors of the selected studies were contacted directly. Initially, all articles were entered based on their titles and abstracts. Subsequently, the complete texts of all shortlisted articles were assessed. Any studies failing to satisfy at least one of the established eligibility criteria were excluded. Ultimately, inclusion was based solely on predetermined selection criteria. From all articles, the following information was extracted: authors' names, publication year, study design, sample size, mean patient age, accelerated method type, and the rate of canine retraction.

Assessment of Risk of Bias in Individual Studies

Two reviewers (AMHA and MYH) assessed the quality of the included studies using the Cochrane Risk of Bias Tool RoB-2 [25]. When there was a disagreement, the third author (AOO) was consulted to reach an agreement. The following domains were rated as low, high, or with some ‘concerns of bias’: risk of bias from the randomization process, risk of bias from deviations from intended interventions, missing outcome data, risk of bias from outcome measurement, and risk of bias from the selection of reported outcomes. The overall risk of bias of the included studies was assessed as follows: (i) low risk of bias, if all fields were assessed as low risk of bias; (ii) some concerns about bias, if at least one or more fields were assessed as some concerns of bias; (iii) high risk of bias, if at least one or more fields were assessed as high risk of bias.

Quality of Evidence

The strength of evidence for each outcome was estimated by the Grading of Recommendations Assessment, Development, and Evaluation (GRADE) assessment [26]. For each examined outcome, the GRADE framework evaluated key factors, including the number of contributing studies, potential risk of bias, inconsistency in results, evidence indirectness, and result imprecision. Two reviewers (AMHA and MYH) performed these assessments independently, with any discrepancies resolved through discussion with a third reviewer (AOO).

Summary Measures, Approach to Synthesis and Analysis

A quantitative synthesis of the impact of LLLT and vibrational stimulation on orthodontic parameters was performed through a meta-analysis using Review Manager (RevMan) version 5.4 (The Cochrane Collaboration, Copenhagen, Denmark), including canine retraction rate, rotation, tipping, and anchorage loss. Only RCTs were included. Data extracted from each study included the mean values, standard deviations (SDs), and sample sizes (N) for both the experimental and control cohorts. The primary metric across most analyses was the monthly rate of canine retraction (mm), while secondary parameters encompassed canine rotation (in degrees), tipping, and anchorage loss (mm). Meta-analyses were carried out using either fixed-effects or random-effects models, contingent upon the degree of heterogeneity, which was evaluated using I² and Chi-square statistics. Outcomes were expressed as mean differences (MDs), along with 95% confidence intervals (CIs). Forest plots were generated for each comparison, and, where applicable, sensitivity analyses were performed to assess the stability of pooled estimates. Furthermore, funnel plots were employed to visually inspect the potential for publication bias. Asymmetry in the distribution of study estimates was interpreted cautiously, and the funnel plot was considered alongside the number of included studies and their variability in sample size and effect direction.

Results

Literature Search Flow

**Figure 1:**
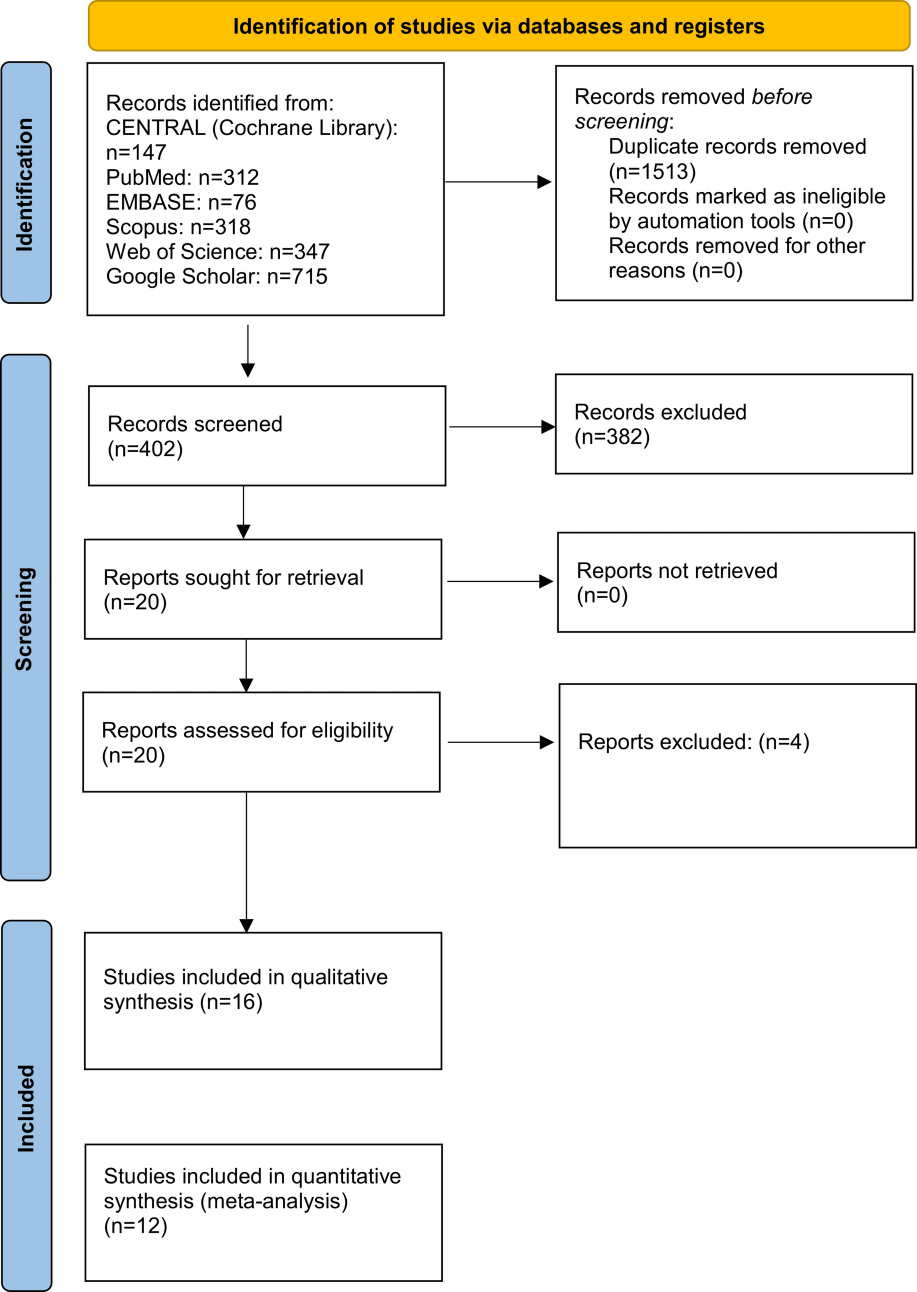
Preferred Reporting Items for Systematic Reviews and Meta-Analysis (PRISMA) flow diagram of the included studies

A comprehensive electronic search across various databases and reference sources identified 1,915 references. After eliminating duplicate records, 402 references underwent a comprehensive evaluation. Title and abstract screening excluded 382 documents, leaving 20 full-text records for further eligibility assessment. Ultimately, the systematic review included 16 studies [14,16-19,27-37]. Twelve studies were included in the quantitative data synthesis. Appendix 2 presents the four studies excluded following full-text evaluation, along with the corresponding reasons for their exclusion. Figure 1 depicts the PRISMA flow diagram outlining the study selection and inclusion procedures.

Characteristics of the Included Studies

Table 3 summarizes the features of the 16 included trials [14,16-19,27-37]. All of these were RCTs published between 2012 and 2024. The included trials were conducted in seven countries, including India [27,28,30-32,37], Egypt [16-18], Australia [33,35], Turkey [29,36], the USA [19], Syria [14], and Japan [34]. Overall, 322 participants were included in these 16 trials, 74% of whom were females. Specifically, 14 out of the 16 trials involved participants of both genders, while two focused exclusively on female patients [17,18]. The findings demonstrated variability in sample sizes (10-45 patients) and ages (14-34 years).

**Table 3:** Characteristics of the included trials EG: experimental group; CG: control group; RCT: randomized controlled trial; SMD: split mouth design; mm/mo: millimeter per month; PGD: parallel group design; NA: not applicable; LLLT: low-level laser therapy; GaAlAs: gallium aluminum arsenide; LPT: light-emitting diode-mediated photobiomodulation therapy; InGaAs: indium gallium arsenide; PEMF: pulsed electromagnetic field therapy; mT: millitesla; SMF: static magnetic field; NdFeB: neodymium iron boron

Author, year, and country	Study design	Patients			Type of interventions/application protocol and timing
		Sample size	Gender: M/F	Age	
Doshi-Mehta and Bhad-Patil (2012) [28]; India	RCT SMD	N = 20	N = 8/12	12-23	- LLLT (GaAlAs 810 nm diode laser) - 5 buccal and 5 palatal points - on days 0, 3, 7, and 14 in the first month, then every 15 days until the end of canine retraction
Pavlin et al. (2015) [19]; USA	RCT PGD	N = 45, EG = 23, CG = 22	NA	12-40	- Vibrations via AcceleDent® device, 0.25 N and 30 Hz - 20 minutes per day until the end of canine retraction
Ekizer et al. (2016) [29]; Turkey	RCT SMD	N = 20	N = 7/13	13-19, Mean: 16.77 ± 1.4	- LPT (618 nm diode laser) - one buccal point - once/day for 21 consecutive days
Abd-El-Ghafour Omar et al. (2017) [16]; Egypt	RCT SMD	N = 22	N = 3/19	18-25, Mean: 21.4 ± 3.2	- LLLT (InGaAs 940 nm diode laser) - one buccal point - once/week at the first 4 weeks, then once every 2 weeks for 3 months
Kochar et al. (2017) [32]; India	RCT SMD	N = 20	N = 12/8	16-24, Mean: 19.69 ± 1.4	- LLLT (810 nm diode laser) - 5 buccal and 5 palatal points - on days 3, 7, and then every 21 days until the end of canine retraction
Liao et al. (2017) [33]; Australia	RCT SMD	N = 13	NA	12.1-15.5; Mean: 13.6	- Oral B Hamming Bird Vibrating Unit, 0.2 N and 50 Hz - 10 minutes per day for 28 consecutive days
Üretürk et al. (2017) [36]; Turkey	RCT SMD	N = 15	N = 7/8	Mean: 16.2 ± 1.3	- LLLT (GaAlAs 810 nm diode laser) - 5 buccal and 5 palatal points - on day 0, 3, 7, 14, 21, 30, 33, 37, 44, 51, 60, 63, 67, 74, 81, 84, 90
Varella et al. (2018) [37]; India	RCT SMD	N = 10	N = 4/6	14-25	- LLLT (GaAlAs 940 nm diode laser) - 5 buccal and 5 palatal points - 3 consecutive days at the start of canine retraction, 4 weeks later, and 8 weeks later
Jivrajani and Bhad-Patil (2020) [30]; India	RCT SMD	N = 10	N = 3/7	14-24	- LLLT (GaAlAs 980 nm diode laser) - 5 buccal and 5 palatal points - on days 1, 3, 5, 7, 14, and then 15 consecutive days
Mistry et al. (2020) [35]; Australia	RCT SMD	N = 21	N = 7/14	13-25, Mean: 17.4 ± 2.6	- LLLT (GaAlAs 808 nm diode laser) - 4 buccal and 4 palatal points - on days 0, 28, and 56
Bhad-Patil and Karemore (2020) [27]; India	RCT SMD	N = 19	N = 2/17	18-24	- PEMF 0.5 mT - removable appliance (8-10) hours per day until the end of canine retraction
Mohamed Hasan et al. (2021) [17]; Egypt	RCT SMD	N = 15	N = 0/15	18-25	- LLLT (910 nm diode laser) - one buccal point - on days 1, 3, 14, and then every 14 days until the end of canine retraction
Mayama et al. (2022) [34]; Japan	RCT SMD	N = 23	N = 4/19	13.5-40.4, Mean: 20.2 ± 7	- Vibrations via AcceleDent® device, 0.05 N, 102 Hz - 3 minutes at monthly visit
Kharat et al. (2023) [31]; India	RCT SMD	N = 20	NA	18-35	- LLLT (GaAlAs 940 nm diode laser) - 1 buccal and 1 palatal points - on days 0, 3, 7, and 14 of the first month, then every 15 days until the end of canine retraction
Abd ElMotaleb et al. (2024) [18]; Egypt	RCT PGD	N = 32, EG = 16, CG = 16	N = 0/32	15-21	- Vibrations via AcceleDent® device, 0.25 N, 30 Hz - 20 minutes per day until the end of canine retraction
Alqaisi et al. (2024) [14]; Syria	RCT SMD	N = 17	N = 4/13	18-28, Mean: 20.76 ± 2.9	- SMF (NdFeB 414 mT magnets) - a fixed appliance remains throughout the retraction phase

Of the studies with specific design types, 14 used a split-mouth design [14,16,17,27-37], while two used a parallel-group design [18,19]. For the types of malocclusion, 10 studies were conducted on Class II patients [17-19,29,30,33-37], three addressed bimaxillary dentoalveolar protrusion [14,27,32], and three included both types [16,28,31]. Regarding the method of physical acceleration, 10 trials applied LLLT [16,17,28-32,35-37], four used low-frequency vibrations [18,19,33,34], and two used electromagnetic field therapy [14,27]. All 16 studies included in this review used nickel-titanium closed-coil springs for canine retraction; 14 used a force of 150 grams [14,16-18,27-33,35-37], one applied 180 grams [19], and another applied 100 grams [34].

There are different approaches to canine retraction assessment; 14 studies designed 3D digital models based on 3D scans of plaster casts using 3D modeling software [14,17,18,28,29,34-37]. Six trials employed manual measurement techniques, utilizing digital calipers to accurately record distances on physical dental casts [19,27,30-33]. Additionally, one trial utilized two-dimensional scans of dental casts, offering a less detailed yet functional approach for analyzing tooth movement [16]. The follow-up period across trials varied widely, ranging from two to seven months. Notably, 13 studies reported follow-up durations of three to five months [14,16-19,28-33,35,36], while one study had an observation time of two months [37], one had six months [27], and the final study had seven months [34].

Risk of Bias for the Included Trials

**Figure 2:**
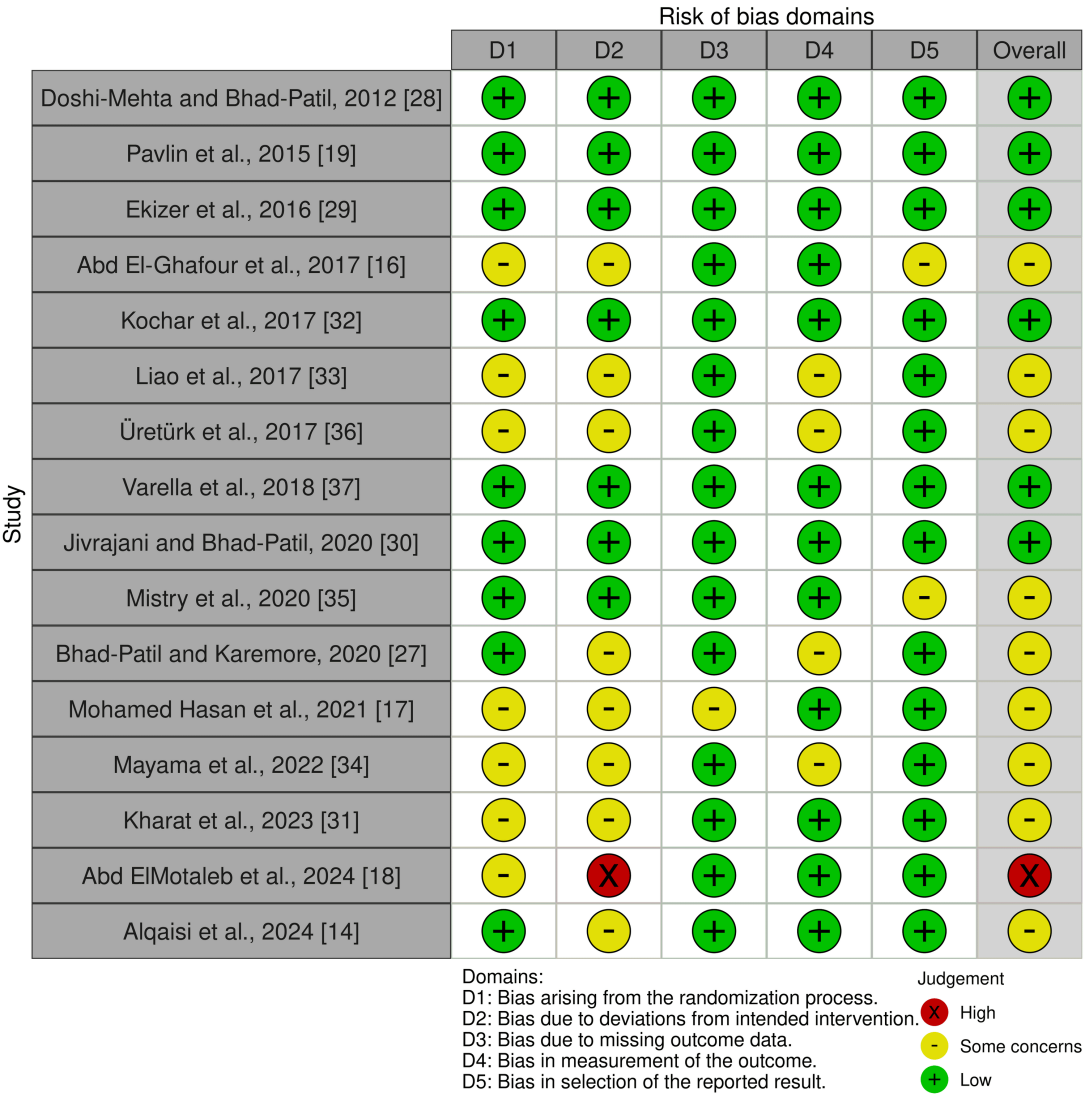
Summary of the risk of bias for each included randomized controlled trial according to the Cochrane RoB 2 tool presented as authors’ judgments across all domains

**Figure 3:**
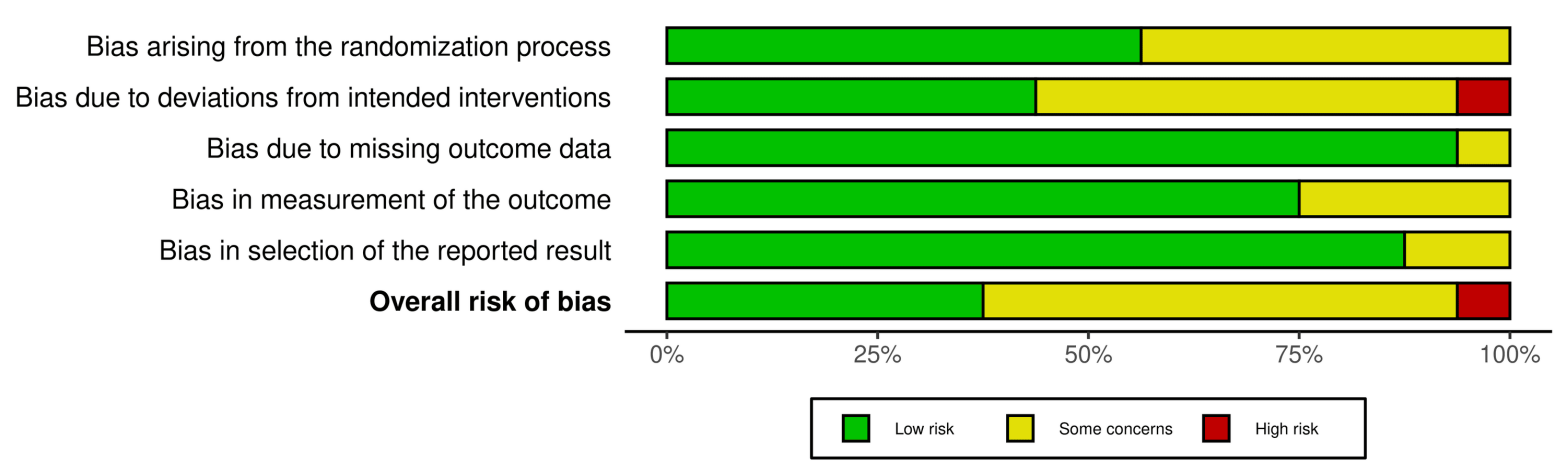
Overall risk of bias assessment across all included studies showing the proportion of trials categorized as low risk, some concerns, or high risk

Six RCTs demonstrated a low risk of bias [19,28-30,32,37], indicating rigorous methodology. Meanwhile, nine RCTs were categorized as having some concerns of bias [14,16,17,27,31,33-36], suggesting potential limitations in study design or execution. One RCT was classified as high risk of bias [18], highlighting notable methodological weaknesses that could impact the reliability of its findings. Figures 2-3 provide a comprehensive summary of the overall risk of bias across the included trials, while Appendix 3 details the specific factors influencing each assessment.

Effects of Interventions

A summary of the main findings of the included studies is given in Table 4.

**Table 4:** Main findings of the included studies CG: control group; EG: experimental group; HS: highly significant; NA: not applicable; ND: no difference; S: significant; NS: not significant

Author, year, and country	Rate of canine retraction	Tipping	Rotation	Anchorage loss	Root resorption
Doshi-Mehta and Bhad-Patil (2012) [28]; India	CG: 0.84 ± 0.21 mm/mo; EG: 1.17 ± 0.22 mm/mo, 40% more than CG HS	NA	NA	NA	NA
Pavlin et al. (2015) [19]; USA	CG: 0.79 ± 0.15 mm/mo; EG: 1.16 ± 0.15 mm/mo, 47% more than CG HS	NA	NA	NA	NA
Ekizer et al. (2016) [29]; Turkey	CG: 0.92 ± 0.67 mm/mo; EG: 1.25 ± 0.63 mm/mo, 36% more than CG HS	NA	NA	NA	NA
Abd-El-Ghafour Omar et al. (2017) [16]; Egypt	CG: 0.95 ± 0.23 mm/mo; EG: 0.95 ± 0.33 mm/mo, Same with CG ND	NA	NA	CG: 1.18 ± 1.54 mm; EG: 1.01 ± 1.41 mm, 18% more than EG NS	NA
Kochar et al. (2017) [32]; India	CG: 0.94 ± 0.014 mm/mo; EG: 1.92 ± 0.16 mm/mo, 104% more than CG HS	NA	NA	NA	NA
Liao et al. (2017) [33]; Australia	CG: 0.87 ± 0.30 mm/mo; EG: 1.32 ± 0.30 mm/mo, 51% more than CG HS	NA	NA	CG: 1.05 mm; EG: 0.56 mm, 88% more than EG HS	NA
Üretürk et al. (2017) [36]; Turkey	CG: 0.92 ± 0.49 mm/mo; EG: 1.3 ± 0.47 mm/mo, 42% more than CG HS	NA	NA	NA	NA
Varella et al. (2018) [37]; India	CG: 1.01 ± 0.30 mm/mo; EG: 2.22 ± 0.30 mm/mo, 120% more than CG HS	NA	NA	NA	NA
Jivrajani and Bhad-Patil (2020) [30]; India	CG: 0.96 ± 0.33 mm/mo; EG: 1.33 ± 0.48 mm/mo, 39% more than CG HS	NA	NA	NA	NA
Mistry et al. (2020) [35]; Australia	CG: 0.82 ± 0.30 mm/mo; EG: 0.91 ± 0.26 mm/mo, 11% more than CG NS	NA	CG: 29.20° ± 9°; EG: 33.21° ± 10.71°, 14% higher than CG NS	CG: 0.36 ± 0.78 mm; EG: 0.66 ± 0.78 mm, 84% more than CG NS	NA
Bhad-Patil and Karemore (2020) [27]; India	CG: 0.82 ± 0.26 mm/mo; EG: 1.45 ± 0.31 mm/mo, 77% more than CG HS	NA	NA	NA	NA
Mohamed Hasan et al. (2021) [17]; Egypt	CG: 0.6 ± 0.47 mm/mo; EG: 1.15 ± 0.53 mm/mo, 92% more than CG HS	CG: 4.34° ± 4.22°; EG: 5.28° ± 4.34°, 22% more than CG NS	CG: 11.91° ± 6.36°; EG: 11.78° ± 6.47°, 1% more than EG NS	CG: 2.11 ± 0.85 mm; EG: 1.42 ± 0.55 mm, 49% more than EG S	NA
Mayama et al. (2022) [34]; Japan	CG: 0.89 ± 0.55 mm/mo; EG: 1.21 ± 0.60 mm/mo, 36% more than CG S	NA	NA	NA	ND
Kharat et al. (2023) [31]; India	CG: 0.74 ± 0.04 mm/mo; EG: 0.81 ± 0.03 mm/mo, 10% more than CG S	NA	NA	NA	NA
Abd ElMotaleb et al. (2024) [18]; Egypt	CG: 1.27 ± 0.92 mm/mo; EG: 1.35 ± 0.75 mm/mo, 6% more than CG NS	CG: 11.3° ± 5.5°; EG: 10.2° ± 4.1°, 11% more than CG NS	CG: 15.1° ± 10°; EG: 12° ± 7.9°, 26% more than EG NS	NA	CG: 0.6 ± 1 mm; EG: 0.8 ± 0.7 mm, 33% more than CG NS
Alqaisi et al. (2024) [14]; Syria	CG: 1.35 ± 0.41 mm/mo; EG: 1.67 ± 0.43 mm/mo, 23.7% more than CG S	NA	NA	CG: 0.41 ± 0.30 mm; EG: 0.32 ± 0.23 mm, 28% more than EG NS	NA

**Figure 4:**
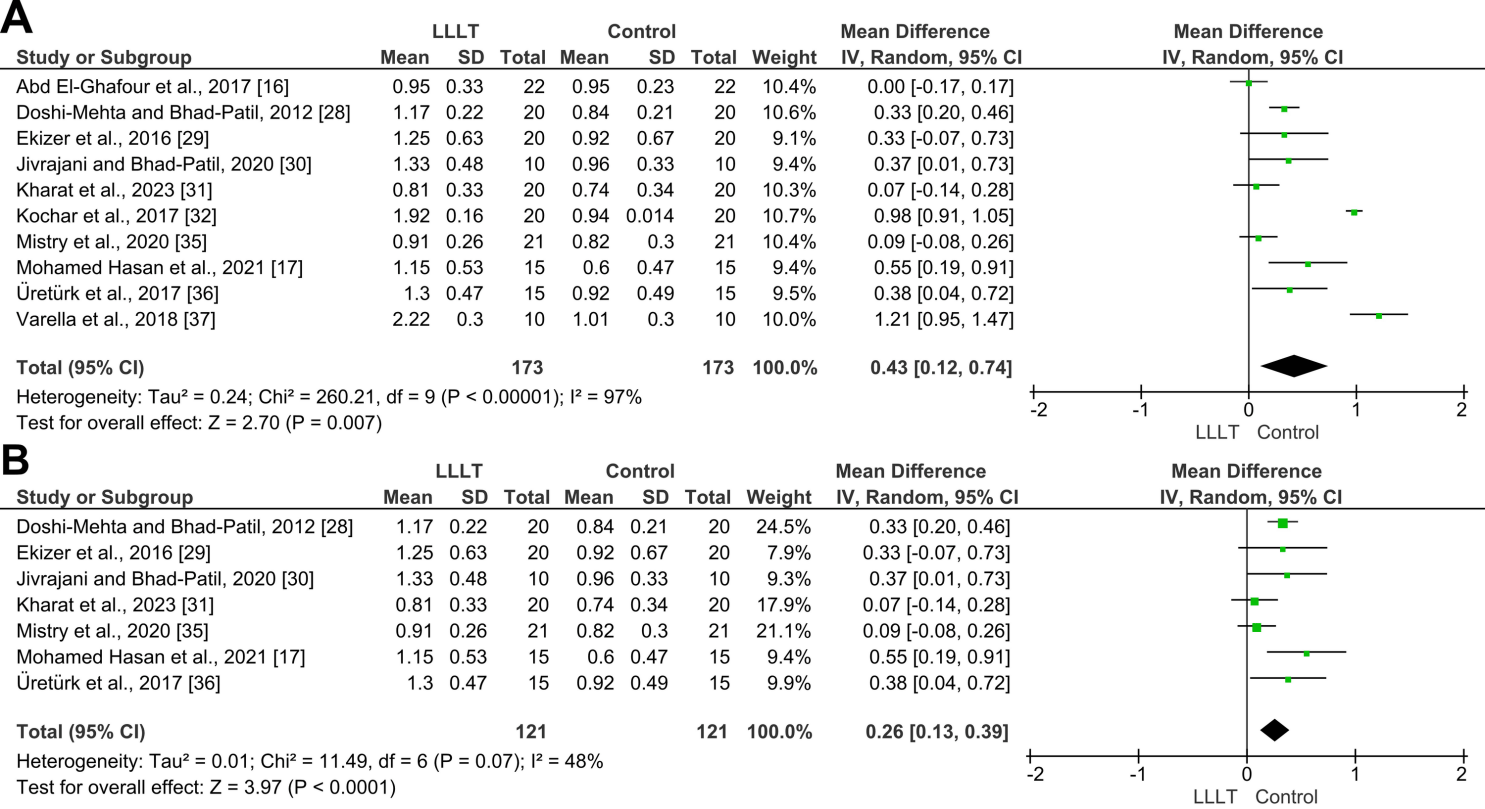
A forest plot depicting the comparative analysis of upper canine retraction rates between the LLLT group and the control group (mm/month) A) Before sensitivity analysis; B) After sensitivity analysis. LLLT: low-level laser therapy

Rate of canine retraction: Among the 10 LLLT trials, retraction rates in experimental groups ranged from 0.81 mm/month in the Kharat et al. [31] trial to 2.22 mm/month in the Varella et al. [37] trial, while control group rates ranged from 0.6 mm/month [17] to 1.01 mm/month [37]. Conducting a meta-analysis of the LLLT studies within a random-effects model showed the retraction rate in the experimental groups to be significantly increased, with an MD of 0.43 mm/month (95% CI: 0.12, 0.74, p = 0.007; Figure 4A). However, the heterogeneity was very high (I² = 97%), indicating substantial variability among the included studies.

To remedy this, a sensitivity analysis was performed by excluding the three most influential studies (Kochar et al. [32], Varella et al. [37], and Abd-El-Ghafour Omar et al. [16]). The updated meta-analysis of the remaining seven studies showed a reduced MD of 0.26 mm/month (95% CI: 0.13, 0.39, p < 0.0001; Figure 4B), with heterogeneity decreasing to an acceptable level (I² = 48%). These findings confirm that while LLLT significantly improves the rate of canine retraction, a few high-impact studies contribute disproportionately to the overall effect size and variability. According to GRADE, the overall strength of the evidence supporting this outcome was low (Table 5).

**Table 5:** Summary of the findings according to the GRADE guidelines for included studies RCTs: randomized controlled trials; LLLT: low-level laser therapy; GRADE: Grading of Recommendations Assessment, Development, and Evaluation ^a^ Downgrade one level for risk of bias, and one level for publication bias, ^b ^Downgrade one level for risk of bias, ^c ^Downgrade one level for risk of bias, and one level due to inconsistency.

Quality assessment criteria						Summary findings			Comments
Number of studies	Risk of bias	Inconsistency	Indirectness	Imprecision	Other consideration	Effect		Certainty	
						Number of patients	Absolute (95% CI)		
Rate of upper canine retraction (LLLT vs Control)									
7 RCTs	Serious	Not Serious	Not Serious	Not Serious	Possible Publication Bias	242	0.26 (0.13, 0.39)	⊕⊕⊖⊖^a^ Low	Significant increase of 0.26 mm in the rate of upper canine retraction with LLLT compared to the control group (p < 0.0001)
Rate of upper canine retraction (Vibration vs Control)									
2 RCTs	Serious	Not Serious	Not Serious	Not Serious	None	77	0.36 (0.28, 0.45)	⊕⊕⊕⊖^b^ Moderate	Significant increase of 0.36 mm in the rate of upper canine retraction with vibration compared to the control group (p < 0.00001)
Upper canine rotation (LLLT vs Control)									
2 RCTs	Serious	Not Serious	Not Serious	Not Serious	None	72	1.48 (-2.48, 5.43)	⊕⊕⊕⊖^b^ Moderate	The difference was not statistically significant (p = 0.46)
Anchorage loss (LLLT vs Control)									
3 RCTs	Serious	Serious	Not Serious	Not Serious	None	116	-0.16 (-0.48, 0.17)	⊕⊕⊖⊖^c^ Low	The difference was not statistically significant (p = 0.34)

The highest reported increases in the rate of canine retraction were observed in two studies not included in the analysis, Kochar et al. [32] and Varella et al. [37]. In Kochar et al., an 810 nm GaAlAs diode laser was applied at five buccal and five palatal points, administered on days 3 and 7 and then every 21 days throughout the retraction phase, resulting in a retraction rate of 1.92 mm/month, a 104% increase over the control group [32]. Varella et al. used a 940 nm GaAlAs diode laser with three consecutive applications at the start of retraction, followed by additional sessions at weeks 4 and 8. This protocol led to a retraction rate of 2.22 mm/month, representing a 120% improvement over controls [37].

**Figure 5:**
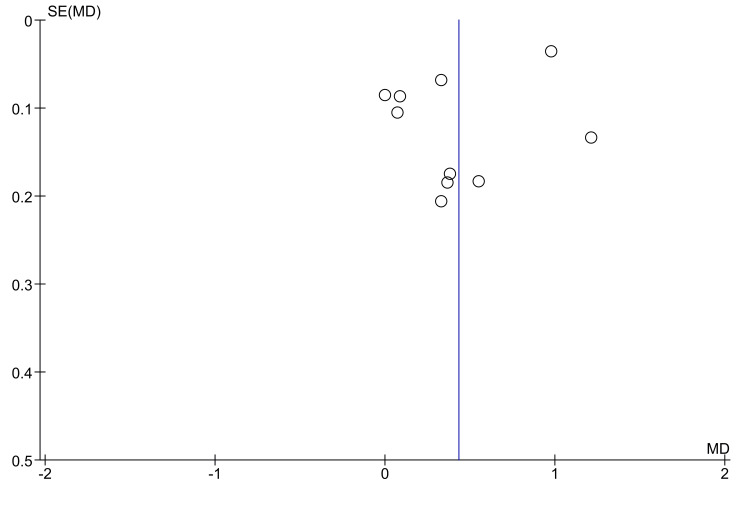
A funnel plot evaluating the potential presence of publication bias in LLLT trials LLLT: low-level laser therapy

In contrast, the third excluded trial by Abd-El-Ghafour Omar et al. [16] reported identical retraction rates for both groups (0.95 mm/month). Figure 5 shows the funnel plot used to analyse potential publication bias. Visual inspection of the plot revealed slight asymmetry, with most studies clustered on the right side of the MD axis and a relative scarcity of studies with small sample sizes reporting negative or null effects. This pattern may suggest publication bias, in which studies with favorable outcomes are more likely to be published or included; therefore, we downgraded the certainty of the evidence by one level.

**Figure 6:**
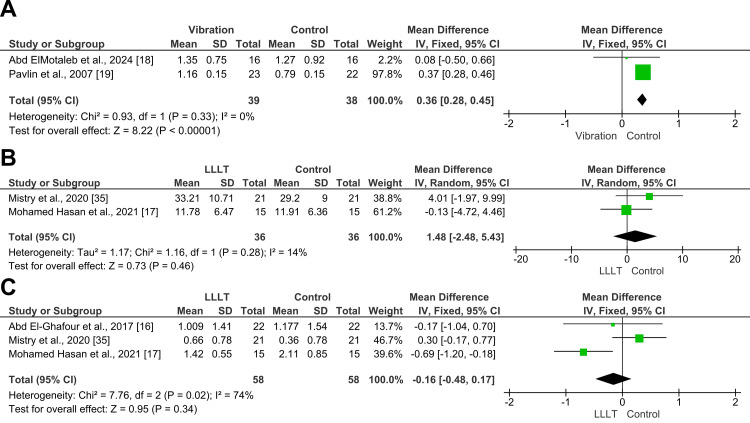
Forest plots of secondary outcomes in this systematic review A) Rate of upper canine retraction between vibration-assisted therapy and control groups (mm/month), B) Canine rotation between LLLT and control groups (degrees), C) Anchorage loss between LLLT and control groups (mm). LLLT: low-level laser therapy

Four trials evaluated vibrational stimulation employing different devices and protocols to enhance canine retraction [18,19,33,34]. A meta-analysis was performed on the two studies by Pavlin et al. [19] and Abd ElMotaleb et al. [18] because both used the AcceleDent® device according to an identical protocol, delivering 0.25 N of force at 30 Hz for 20 minutes per day throughout the retraction phase. The analysis revealed a statistically significant acceleration in the retraction rate in the vibration group compared to the control, with an MD of 0.36 mm/month (95% CI: 0.28, 0.45, p < 0.00001; Figure 6A). Notably, no heterogeneity was detected (I² = 0%), indicating consistent results across both trials. According to GRADE, the overall strength of the evidence supporting this outcome was moderate.

Liao et al. [33] applied the Oral-B HummingBird Vibrating Unit (0.2 N, 50 Hz) for 10 minutes daily over 28 consecutive days, achieving a retraction rate of 1.32 mm/month - a 51% acceleration over the control group (0.87 mm/month). Mayama et al. [34] utilized AcceleDent® with a different protocol, delivering 0.05 N at 102 Hz for three minutes at each monthly visit, resulting in a retraction rate of 1.21 mm/month - a 36% increase compared to the control group (0.89 mm/month). Both studies demonstrated significant enhancements in retraction speed, reinforcing the efficacy of vibration-based interventions.

Two studies have used electromagnetic field therapy. Bhad-Patil and Karemore applied PEMF therapy and reported an increase from 0.82 mm/month in the control group to 1.45 mm/month in the experimental group [27]. Alqaisi et al. applied an SMF [14], reporting a retraction rate of 1.67 mm/month, compared with 1.35 mm/month in the control group. The observed acceleration in these two studies ranged from 19% to 77%. Overall, experimental interventions were associated with higher rates of canine retraction than conventional methods.

Canine angulation (canine tip): Two trials investigated the change in canine angulation after retraction [17,18]. One trial used LLLT, and the other used low-frequency vibration. Both trials found that canine angulation increased after retraction, but the differences between the experimental and control groups were not statistically significant.

Canine rotation: Three trials examined rotational changes in the canines during retraction [17,18,35]. Among them, two trials implemented LLLT [17,35]. When these LLLT studies were pooled in a meta-analysis, the overall effect was non-significant, with an MD of 1.48° (95% CI: -2.48, 5.4, p = 0.46; Figure 6B). Heterogeneity was low (I² = 14%), indicating consistency in findings, but the combined result suggests that LLLT does not significantly affect canine rotation. According to GRADE, the overall strength of the evidence supporting this outcome was moderate. The third trial employed low-frequency vibration [18] and found that the control group exhibited a marginally greater rotational change in canines, though the difference was not statistically significant.

Canine root resorption following retraction: Only one trial investigated this area [18]. It utilized vibrational forces with the AcceleDent Aura device and found that canine root resorption after retraction was slightly higher in the experimental group, though the difference was not statistically significant.

Anchorage loss following retraction: Five trials investigated the mesial movement of the upper first molars during canine retraction [14,16,17,33,35]. Of these, three studies evaluated the effects of LLLT [16,17,35]. A meta-analysis of these three studies showed no statistically significant overall effect of LLLT on anchorage loss, with a pooled MD of -0.16 (95% CI: -0.48 to 0.17; p = 0.34; Figure 6C), indicating that LLLT did not significantly reduce anchorage loss compared to the control. Substantial heterogeneity was observed among the included studies (I² = 74%, p = 0.02). According to GRADE, the overall strength of the evidence supporting this outcome was low. The fourth trial assessed low-frequency vibration and reported a highly significant finding: anchorage loss in the control group was nearly twice that of the experimental group [33]. Meanwhile, the fifth trial examined a low-intensity SMF and found no significant difference between the groups [14].

Discussion

This systematic review and meta-analysis highlight how physical acceleration techniques, such as LLLT, mechanical vibrations, and electromagnetic fields, can improve the rate of maxillary canine retraction. The effectiveness, however, is known to vary widely across studies. Many factors contribute to this, such as varying protocols, laser settings, and patient demographics. These findings help clinicians pinpoint the patient populations most likely to benefit from such treatments, thereby facilitating the creation of more customized and efficient strategies. This review strengthens the evidence-based decision-making foundation for orthodontists by synthesizing high-quality RCTs and providing a more robust rationale for adopting evidence-based practices. Furthermore, it highlights key gaps in the current literature, including limited long-term outcome data and a lack of protocol standardization. These insights point to future research directions to refine and validate these techniques for broader, more consistent clinical application.

Rate of Canine Retraction

Low-level laser therapy (LLLT): LLLT emerged as the most frequently studied modality, with 10 included trials. The meta-analysis showed that LLLT significantly increased the canine retraction rate compared to controls, with an MD of 0.43 mm/month. However, the observed heterogeneity (I² = 97%) was substantial, which necessitated a sensitivity analysis. When three influential studies - Kochar et al. [32], Varella et al. [37], and Abd-El-Ghafour Omar et al. [16] - were excluded, the adjusted MD decreased to 0.26 mm/month, and heterogeneity was reduced (I² = 48%). This underscores how a few studies with high acceleration values may have inflated the overall estimate. According to the GRADE assessment, the overall certainty of the evidence for the effect of LLLT on the rate of canine retraction was judged to be low, primarily due to concerns about risk of bias in the included trials and suspected publication bias. This means that our confidence in the estimated effect is limited, and the true effect may be substantially different. Additional well-designed and transparently reported RCTs are required to strengthen the evidence base.

The variation in laser parameters may explain this inconsistency. Wavelengths ranged from 618 nm to 980 nm, with variable application protocols - from single-point buccal applications to multiple bucco-palatal points - and differences in session frequency and duration. For instance, Varella et al. [37] employed high-frequency sessions across multiple sites and observed the greatest acceleration, suggesting that more intensive protocols may yield better outcomes. In contrast, Abd-El-Ghafour Omar et al. [16], who used less frequent weekly and biweekly applications, reported no significant difference between the test and control groups. These findings emphasize the urgent necessity of protocol standardization for LLLT in future research endeavors.

Although the pooled effect sizes observed in this review (0.26-0.43 mm/month) may appear modest, their potential cumulative impact should not be underestimated. For example, even a difference of approximately 0.3 mm/month could shorten the canine retraction phase by two to three months, which is clinically meaningful in the context of orthodontic treatment that often extends over several years. Shorter treatment duration may translate into fewer visits, lower costs, and improved patient satisfaction and compliance, as well as reduced risks associated with prolonged fixed-appliance therapy (e.g., caries, gingival inflammation, and root resorption).

Vibrational stimulation: Vibrational therapy also has positive effects. The studies that followed the same AcceleDent® protocols (Pavlin et al. [19] and Abd ElMotaleb et al. [18]) yielded consistent and statistically significant results, with an MD of 0.36 mm/month and no heterogeneity (I² = 0%). These findings suggest that when vibration parameters (force, frequency, and duration) are standardized, results are more reproducible.

Other studies, such as those by Liao et al. [33] and Mayama et al. [34], used different devices and protocols. Liao et al., for example, used a higher-frequency unit of 50 Hz for 10 minutes a day and reported a 51% acceleration in outcomes compared with controls [33]. Mayama et al.’s approach involved very short-duration sessions (3 minutes/month) with moderate success. These results illustrate the importance of not only the device but also the timing and intensity of application in treatment outcomes. As per the GRADE evaluation, the strength of evidence for the vibration-assisted acceleration of canine retraction was also moderate, with consistent results, low heterogeneity, and acceptable study quality.

Despite the positive findings, questions remain about the biological mechanism by which vibrations enhance tooth movement. Some authors hypothesize that cyclic mechanical loading may stimulate osteoclastic activity and bone remodeling, but more histological studies are needed to confirm this pathway in humans.

Electromagnetic field applications: Electromagnetic therapies, such as PEMF and SMF, have attracted interest from researchers, as they have been examined in only two trials [14,27]. Both studies reported moderate improvements in canine retraction rates, with acceleration ranging from 19% to 77%. Despite their promise, the lack of additional studies confirming these findings limits the scientific strength of these results.

The biological plausibility of electromagnetic acceleration is supported by in vitro evidence showing increased cellular activity under electromagnetic influence. However, translating these findings into consistent clinical benefits requires more robust and standardized clinical trials.

Canine Angulation (Canine Tip)

The angular change of canine crowns during retraction was assessed in two studies [17,18], one of which employed LLLT and the other vibrational stimulation. Neither study reported statistically significant differences in tipping between experimental and control groups. This indicates that, while physical acceleration techniques may increase the speed of retraction, they do not appear to reduce control over tooth angulation.

Maintaining proper angulation is essential in orthodontic biomechanics, as excessive tipping can compromise occlusal and esthetic outcomes. The findings here are reassuring, indicating that acceleration does not necessarily come at the expense of controlled tooth movement.

Canine Rotation

Three studies examined canine rotation, with two using LLLT [17,35] and one using vibrational therapy [18]. The pooled analysis of the LLLT studies showed no statistically significant effect, with an MD of 1.48° and low heterogeneity. This indicates that LLLT does not considerably alter the degree of rotational control during canine movement. This conclusion is supported by the GRADE analysis, which rated the strength of evidence for this outcome as moderate, given the consistency of findings across trials and low statistical heterogeneity.

The third trial, which investigated vibration, showed slightly greater rotational changes in the control group; however, the difference was not significant. These findings are consistent with the notion that physical acceleration methods target translational speed rather than the rotational axis of motion.

Although rotation control is often overlooked in studies of movement speed, it remains critical for optimal root positioning. Future studies should incorporate digital 3D superimposition techniques to more accurately assess subtle rotational differences.

Root Resorption

Only one included trial assessed root resorption following vibrational therapy [18]. The study found a slight increase in resorption in the experimental group, though the difference was not statistically significant. Given that root resorption is a multifactorial phenomenon influenced by force magnitude, duration, and individual predisposition, attributing changes solely to the acceleration modality is challenging.

While the absence of significant findings offers some reassurance, the limited available data prevents drawing definitive conclusions. This area remains underexplored in the literature and represents a critical gap that future studies should address, particularly through advanced imaging modalities such as cone beam computed tomography (CBCT), which offer higher resolution than conventional radiographs, despite the ethical considerations that limit their widespread use in research investigating the side effects of orthodontic acceleration on root resorption.

Anchorage Loss

Anchorage preservation is a critical concern in canine retraction, as mesial movement of the first molars can offset the benefits of accelerated anterior movement. Five studies assessed anchorage loss, with varying results [14,16,17,33,35].

Meta-analysis of the three LLLT studies revealed no significant effect on molar anchorage preservation (MD = -0.16 mm, p = 0.34), although heterogeneity was substantial (I² = 74%) [16,17,35]. This suggests variability in how LLLT affects anchorage units, possibly influenced by force vectors or appliance design. According to GRADE, the evidence for LLLT's effect on anchorage preservation was low, mainly due to substantial heterogeneity and imprecision in the results.

Conversely, the study by Liao et al. found a significant difference in favor of the experimental vibration group [33], with anchorage loss reduced by almost half. These findings suggest that certain vibrational protocols may enhance posterior anchorage, perhaps by minimizing molar drift through enhanced bone density or periodontal support.

One study evaluating SMF found no significant effect on anchorage [14], suggesting that this modality may not influence anchorage control in a clinically meaningful way. Given the limited evidence and variable findings, anchorage remains a vital outcome for future standardized trials to monitor rigorously.

Overall, this review supports the utility of physical acceleration techniques, particularly LLLT and mechanical vibration, in enhancing canine retraction. The findings have clinical implications for reducing overall treatment time, improving patient compliance, and potentially decreasing risks associated with prolonged orthodontic therapy, such as caries and gingival inflammation.

Limitations

We have several restrictions to point out. The high variability within laser studies undermines the applicability of the results. In addition, low sample sizes in numerous studies raise concerns regarding statistical power. Another important restriction is the variability in the outcome measures. Some studies utilize 3D models, while others use manual caliper measurements, introducing potential measurement bias. In addition, short follow-up periods, typically three to five months, overlook chronic aspects such as root integrity and the stability of results. There is also a risk of publication bias, as suggested by funnel plot asymmetry. This is important for interpreting results, as studies with null or negative findings are likely to be much more underrepresented than the existing literature suggests.

## Conclusions

This systematic review and meta-analysis suggest that LLLT may accelerate the rate of maxillary canine retraction during orthodontic treatment. However, the certainty of the evidence for this primary outcome was rated as low, due to concerns about risk of bias across the included randomized trials and potential publication bias. For secondary outcomes, such as canine angulation, rotation, and root resorption, the available evidence was very limited and based on only a few small trials, which preclude drawing reliable conclusions. The included studies were predominantly split-mouth in design, often without appropriate adjustment for intra-patient correlation, and exhibited methodological limitations that may have led to overestimation of precision. Considerable heterogeneity was also present across protocols, laser parameters, and follow-up times, further limiting the generalizability of the findings.

Taken together, these issues mean that the results should be interpreted with caution. While LLLT remains a promising adjunctive approach in orthodontics, there is a clear need for further well-designed, adequately powered, and transparently reported RCTs that use standardized protocols and robust analytic methods. Such studies will be essential to provide more definitive evidence on both the efficacy and the safety of LLLT in accelerating orthodontic tooth movement.

## Appendices

Appendix 1

**Table 6:** Electronic search strategy

Database	Search strategy
CENTRAL	#1 Orthodontic treatment OR orthodontic therapy OR orthodontics OR tooth movement OR orthodontic tooth movement OR tooth displacement OR class II malocclusion OR class II division I OR class II division II OR bimaxillary protrusion #2 Canine retraction OR canine distalization OR two-step retraction OR space closure OR extraction therapy OR close coil spring OR power chain elastic #3 Acceleration OR tooth movement acceleration OR rapid OR velocity OR duration OR rate OR regional accelerated phenomenon OR RAP OR Low-level laser therapy OR LLLT OR low-intensity laser therapy OR LILT OR pulsed electromagnetic field OR PEMF OR static electromagnetic field OR SMF OR mechanical vibrations OR vibrational force OR light-emitting diode-mediated photobiomodulation therapy OR LPT #4 #1 AND #2 AND #3
EMBASE	#1 Orthodontic treatment OR orthodontic therapy OR orthodontics OR tooth movement OR orthodontic tooth movement OR tooth displacement OR class II malocclusion OR class II division I OR class II division II OR bimaxillary protrusion #2 Canine retraction OR canine distalization OR two-step retraction OR space closure OR extraction therapy OR close coil spring OR power chain elastic #3 Acceleration OR tooth movement acceleration OR rapid OR velocity OR duration OR rate OR regional accelerated phenomenon OR RAP OR Low-level laser therapy OR LLLT OR low-intensity laser therapy OR LILT OR pulsed electromagnetic field OR PEMF OR static electromagnetic field OR SMF OR mechanical vibrations OR vibrational force OR light-emitting diode-mediated photobiomodulation therapy OR LPT #4 #1 AND #2 AND #3
PubMed	#1 Orthodontic treatment OR orthodontic therapy OR orthodontics OR tooth movement OR orthodontic tooth movement OR tooth displacement OR class II malocclusion OR class II division I OR class II division II OR bimaxillary protrusion #2 Canine retraction OR canine distalization OR two-step retraction OR space closure OR extraction therapy OR close coil spring OR power chain elastic #3 Acceleration OR tooth movement acceleration OR rapid OR velocity OR duration OR rate OR regional accelerated phenomenon OR RAP OR Low-level laser therapy OR LLLT OR low-intensity laser therapy OR LILT OR pulsed electromagnetic field OR PEMF OR static electromagnetic field OR SMF OR mechanical vibrations OR vibrational force OR light-emitting diode-mediated photobiomodulation therapy OR LPT #4 #1 AND #2 AND #3
Scopus	#1 TITLE-ABS-KEY (Orthodontic treatment OR orthodontic therapy OR orthodontics OR tooth movement OR orthodontic tooth movement OR tooth displacement OR class II malocclusion OR class II division I OR class II division II OR bimaxillary protrusion) #2 TITLE-ABS-KEY (Canine retraction OR canine distalization OR two-step retraction OR space closure OR extraction therapy OR close coil spring OR power chain elastic) #3 TITLE-ABS-KEY (Acceleration OR tooth movement acceleration OR rapid OR velocity OR duration OR rate OR regional accelerated phenomenon OR RAP OR Low-level laser therapy OR LLLT OR low-intensity laser therapy OR LILT OR pulsed electromagnetic field OR PEMF OR static electromagnetic field OR SMF OR mechanical vibrations OR vibrational force OR light-emitting diode-mediated photobiomodulation therapy OR LPT) #4 #1 AND #2 AND #3
Web of Science	#1TS= (Orthodontic treatment OR orthodontic therapy OR orthodontics OR tooth movement OR orthodontic tooth movement OR tooth displacement OR class II malocclusion OR class II division I OR class II division II OR bimaxillary protrusion) #2TS= (Canine retraction OR canine distalization OR two-step retraction OR space closure OR extraction therapy OR close coil spring OR power chain elastic) #3TS= (Acceleration OR tooth movement acceleration OR rapid OR velocity OR duration OR rate OR regional accelerated phenomenon OR RAP OR Low-level laser therapy OR LLLT OR low-intensity laser therapy OR LILT OR pulsed electromagnetic field OR PEMF OR static electromagnetic field OR SMF OR mechanical vibrations OR vibrational force OR light-emitting diode-mediated photobiomodulation therapy OR LPT) #4 #1 AND #2 AND #3
Google Scholar	#1 (Orthodontic treatment OR orthodontic therapy OR orthodontics OR tooth movement OR orthodontic tooth movement OR tooth displacement OR class II malocclusion OR class II division I OR class II division II OR bimaxillary protrusion) AND (Canine retraction OR canine distalization OR two-step retraction OR space closure OR extraction therapy OR close coil spring OR power chain elastic) AND (Acceleration OR tooth movement acceleration OR rapid OR velocity OR duration OR rate OR regional accelerated phenomenon OR RAP OR Low-level laser therapy OR LLLT OR low-intensity laser therapy OR LILT OR pulsed electromagnetic field OR PEMF OR static electromagnetic field OR SMF OR mechanical vibrations OR vibrational force OR light-emitting diode-mediated photobiomodulation therapy OR LPT).

Appendix 2

**Table 7:** Excluded studies and the reasons for exclusion

Study	Reason for exclusion
Dalaie K, Hamedi R, Kharazifard MJ, Mahdian M, Bayat M. Effect of low-level laser therapy on orthodontic tooth movement: a clinical investigation. J Dent (Tehran). 2015 Apr; 12(4):249-56. PMID: 26622279; PMCID: PMC4662762.	Canines were retracted using sectional closing loops
Guram G, Reddy RK, Dharamsi AM, Syed Ismail PM, Mishra S, Prakashkumar MD. Evaluation of low-level laser therapy on orthodontic tooth movement: a randomized control study. Contemp Clin Dent. 2018 Jan-Mar; 9(1):105-109. doi: 10.4103/ccd.ccd_864_17. PMID: 29599594; PMCID: PMC5863391.	Canines were retracted using sectional closing loops
Yassaei S, Aghili H, Afshari JT, Bagherpour A, Eslami F. Effects of diode laser (980 nm) on orthodontic tooth movement and interleukin 6 levels in gingival crevicular fluid in female subjects. Lasers Med Sci. 2016 Dec; 31(9):1751-1759. doi: 10.1007/s10103-016-2045-1. Epub 2016 Sep 28. PMID: 27680969.	The article does not provide specific details about the rate of canine retraction
Youssef M, Ashkar S, Hamade E, Gutknecht N, Lampert F, Mir M. The effect of low-level laser therapy during orthodontic movement: a preliminary study. Lasers Med Sci. 2008 Jan; 23(1):27-33. doi: 10.1007/s10103-007-0449-7. Epub 2007 Mar 15. PMID: 17361391.	Canines were retracted using sectional Ricketts springs

Appendix 3

**Table 8:** Reasons beyond the judges regarding the risk of bias of the included RCTs RCT: randomized controlled trial; CBCT: cone beam computed tomography

Study	Randomization process	Deviations from intended interventions	Missing outcome data	Measurement of the outcome	Selection of the reported results	Overall bias judgment
Doshi-Mehta and Bhad-Patil (2012) [28]	Low risk: A split-mouth design was used, and allocation concealment was ensured.	Low risk: Patients, investigators, and operators were blinded.	Low risk: No missing data was reported.	Low risk: Tooth movement was measured using digital calipers (objective).	Low risk: No evidence of selective reporting.	Low risk
Pavlin et al. (2015) [19]	Low risk: Computer-generated randomization was conducted, and allocation concealment was ensured.	Low risk: Patients, investigators, and operators were blinded.	Low risk: Two participants were excluded due to occlusal interference.	Low risk: Movement was measured using CBCT scans and digital calipers (objective).	Low risk: No evidence of selective reporting.	Low risk
Ekizer et al. (2016) [29]	Low risk: Randomization was achieved via coin toss, and concealment was ensured using sealed envelopes.	Low risk: Sham application ensured patient blinding, and the operator was blinded to treatment allocation.	Low risk: No missing data was reported.	Low risk: Movement was measured using 3D cast superimposition, and cytokine levels were evaluated using ELISA.	Low risk: No evidence of selective reporting.	Low risk
Abd-El-Ghafour Omar et al. (2017) [16]	Some concerns: Computer-generated randomization was conducted, but concealment details were unclear.	Some concerns: Patients were unblinded, though assessors were blinded.	Low risk: All 22 randomized participants were analyzed, and minor missing data, 20/242 laser applications were missed due to appointments, but this did not affect the primary outcome analysis.	Low risk: CBCT imaging was used (objective).	Some concerns: No trial registration or pre-published protocol. Unable to verify if all planned outcomes were reported due to a lack of protocol.	Some concerns
Kochar et al. (2017) [32]	Low risk: A split-mouth design was used, and allocation concealment was ensured.	Low risk: Sham laser exposure ensured patient blinding.	Low risk: No missing data was reported.	Low risk: Movement was measured using digital calipers (objective).	Low risk: No evidence of selective reporting.	Low risk
Liao et al. (2017) [33]	Some concerns: A randomized split-mouth design was used, but concealment details were unclear.	Some concerns: Patients were aware of their intervention, and manual variations in vibration application could introduce bias.	Low risk: No missing data was reported.	Some concerns: Movement was measured using digital calipers and model scans (objective), with computational validation to support results.	Low risk: No evidence of selective reporting.	Some concerns
Üretürk et al. (2017) [36]	Some concerns: Split-mouth randomization was conducted, but concealment details were unclear.	Some concerns: Sham laser exposure ensured patient blinding, but operators were aware.	Low risk: No missing data was reported.	Some concerns: Movement was measured using 3D model scans (objective), but cytokine variability could introduce bias.	Low risk: No evidence of selective reporting.	Some concerns
Varella et al. (2018) [37]	Low risk: Sealed envelope allocation was used, and allocation concealment was ensured.	Low risk: Sham laser exposure ensured patient blinding.	Low risk: No missing data was reported.	Low risk: Movement was measured using a digital occlusogram (objective), and cytokine levels were analyzed using ELISA for IL-1β.	Low risk: No evidence of selective reporting.	Low risk
Jivrajani and Bhad-Patil (2020) [30]	Low risk: Allocation was done using chit-based selection, and allocation concealment was ensured.	Low risk: Sham laser exposure ensured patient blinding.	Low risk: No missing data was reported.	Low risk: Movement was assessed using digital calipers (objective), and cytokine levels were analyzed using ELISA.	Low risk: No evidence of selective reporting.	Low risk
Mistry et al. (2020) [35]	Low risk: A triple-blind RCT was conducted, and allocation concealment was ensured using sealed envelopes.	Low risk: Patients, operators, and statistic assessors were blinded.	Low risk: 1/22 participants were excluded due to appliance breakage (attrition balanced, CONSORT flow diagram), and intention-to-treat analysis was performed.	Low risk: Movement was assessed using digital models and calipers (objective), and blinding minimized detection bias.	Some concerns: The protocol was not published prospectively (no pre-registered analysis plan) and no evidence of selective reporting, but unable to be fully verified without the protocol.	Some concerns
Bhad-Patil and Karemore (2020) [27]	Low risk: Sealed envelope randomization was conducted.	Some concerns: PEMF device compliance was self-reported without monitoring.	Low risk: No missing data was reported.	Some concerns: Movement was measured using digital calipers (objective).	Low risk: No evidence of selective reporting.	Some concerns
Mohamed Hasan et al. (2021) [17]	Some concerns: A split-mouth design was used, but the details of allocation concealment were unclear.	Some concerns: Patients were blinded, but operators were aware of treatment allocation.	Some concerns: Three participants were lost to follow-up.	Low risk: Tooth movement was measured using alginate impressions and CBCT scans (objective).	Low risk: No evidence of selective reporting.	Some concerns
Mayama et al. (2022) [34]	Some concerns: A double-blind RCT was conducted, but allocation concealment details were unclear.	Some concerns: Patients and orthodontists were blinded, but compliance outside clinic visits was not monitored.	Low risk: Two patients were excluded due to occlusal interference.	Some concerns: Movement was measured using 3D dentition models (objective), but pain and root resorption were assessed subjectively using radiographs.	Low risk: No evidence of selective reporting.	Some concerns
Kharat et al. (2023) [31]	Some concerns: The split-mouth method was used, but allocation concealment details were unclear.	Some concerns: Patients were blinded, but operator blinding was not possible.	Low risk: No missing data was reported.	Low risk: Tooth movement was measured using digital calipers (objective).	Low risk: No evidence of selective reporting.	Some concerns
Abd ElMotaleb et al. (2024) [18]	Some concerns: Random allocation (1:1 ratio) was used, but concealment details were unclear.	High risk: Patients were aware of the intervention, and compliance was self-reported without verification.	Low risk: No missing data was reported.	Low risk: CBCT imaging provided objective measurements.	Low risk: No evidence of selective reporting.	High risk
Alqaisi et al. (2024) [14]	Low risk: Computer-generated randomization ensured balanced baseline characteristics.	Some concerns: Split-mouth design was used, assessors were blinded, but operators were aware.	Low risk: No missing data was reported.	Low risk: Digital cast analysis ensured objective assessment with minimal bias.	Low risk: No evidence of selective reporting.	Some concerns
